# Ensem-HAR: An Ensemble Deep Learning Model for Smartphone Sensor-Based Human Activity Recognition for Measurement of Elderly Health Monitoring

**DOI:** 10.3390/bios12060393

**Published:** 2022-06-07

**Authors:** Debarshi Bhattacharya, Deepak Sharma, Wonjoon Kim, Muhammad Fazal Ijaz, Pawan Kumar Singh

**Affiliations:** 1 Department of Electronics and Communication Engineering, Techno Main Salt Lake, Salt Lake City, EM-4/1, Sector-V, Kolkata 700091, West Bengal, India; bhattacharyadebarshi4@gmail.com; 2 Department of Information Technology, Jadavpur University Second Campus, Jadavpur University, Plot No. 8, Salt Lake Bypass, LB Block, Sector III, Salt Lake City, Kolkata 700106, West Bengal, India; dkhandelwal4964@gmail.com (D.S.); pksingh.it@jadavpuruniversity.in (P.K.S.); 3 Division of Future Convergence (HCI Science Major), Dongduk Women’s University, Seoul 02748, Korea; 4 Department of Intelligent Mechatronics Engineering, Sejong University, Seoul 05006, Korea

**Keywords:** Human Activity Recognition, Ensem-HAR, elderly health monitoring, deep learning, WISDM, PAMAP2, UCI-HAR

## Abstract

Biomedical images contain a huge number of sensor measurements that can provide disease characteristics. Computer-assisted analysis of such parameters aids in the early detection of disease, and as a result aids medical professionals in quickly selecting appropriate medications. Human Activity Recognition, abbreviated as ‘HAR’, is the prediction of common human measurements, which consist of movements such as walking, running, drinking, cooking, etc. It is extremely advantageous for services in the sphere of medical care, such as fitness trackers, senior care, and archiving patient information for future use. The two types of data that can be fed to the HAR system as input are, first, video sequences or images of human activities, and second, time-series data of physical movements during different activities recorded through sensors such as accelerometers, gyroscopes, etc., that are present in smart gadgets. In this paper, we have decided to work with time-series kind of data as the input. Here, we propose an ensemble of four deep learning-based classification models, namely, ‘CNN-net’, ‘CNNLSTM-net’, ‘ConvLSTM-net’, and ‘StackedLSTM-net’, which is termed as ‘Ensem-HAR’. Each of the classification models used in the ensemble is based on a typical 1D Convolutional Neural Network (CNN) and Long Short-Term Memory (LSTM) network; however, they differ in terms of their architectural variations. Prediction through the proposed Ensem-HAR is carried out by stacking predictions from each of the four mentioned classification models, then training a Blender or Meta-learner on the stacked prediction, which provides the final prediction on test data. Our proposed model was evaluated over three benchmark datasets, WISDM, PAMAP2, and UCI-HAR; the proposed Ensem-HAR model for biomedical measurement achieved 98.70%, 97.45%, and 95.05% accuracy, respectively, on the mentioned datasets. The results from the experiments reveal that the suggested model performs better than the other multiple generated measurements to which it was compared.

## 1. Introduction

Human Activity Recognition (HAR) is one of the most active and fascinating study fields in computer vision and human–computer interaction. In the fields of ubiquitous computing, interpersonal interactions, and human behavior analysis, automatically recognizing human physical activities has become a serious challenge. Because of the enormous advancements in microelectronics over the last decade, several complicated and high computational power devices have been developed capable of doing more complex tasks than ever before. Because of their compact size, low cost, tremendous computing capacity, and low power consumption, these devices have become a part of people’s daily lives. Recognizing human actions, in particular, has been a hot topic in the discipline, particularly for medical, military, and security applications.

Signals obtained in real-time from many body-worn inertial sensors are used to recognize human activities. Sensors such as accelerometers, gyroscopes, and others are utilized in smart devices (often smartphones). The accelerometer, magnetometer, and gyroscope are now standard features on most smartphones. Physical human activity can be recognized by evaluating data collected from numerous smart wearable sensing devices and processed and evaluated using a classification system. Activity prediction is useful because it allows individuals to track their daily routines. Cell phones have become an integral part of our lives, and they are outfitted with sensors that can monitor people’s movements. These sensor data help us to recognize actions such as walking, sleeping, sprinting, sitting, walking downstairs, upstairs, etc. Various sensors are used based on the various actions that we are attempting to forecast based on our requirements. In addition to sensor data, images and videos can be used for the task of recognizing human activities [1].

HAR can be used in various sectors, a few of which are mentioned below.

Monitoring is employed to prevent crimes and deadly terrorist acts from occurring in public places, such as in a system that provides a comprehensive and deployable activity recognition system with on-demand real-time activity recognition based on crowd input from public security surveillance cameras.

HAR is used in numerous sectors, particularly in the residential, hospital, and restructuring sectors. HAR has been integrated with smart devices to keep track of the day-to-day activities of older persons who stay at home or in rehabilitation centres. This helps to keep older people healthy and prevents them from contracting harmful diseases through monitoring of different activities such as heart rate, oxygen levels, calorie intake, calorie burn, and other activities. HAR has become a highly effective way to control and regulate patients’ physical daily activities by following up and keeping track of their devices and activities and maintaining patient health accordingly in order to minimize the risk of a variety of life-threatening diseases such as diabetes, overexposure, and cardio activity [2].

**Figure 1:**
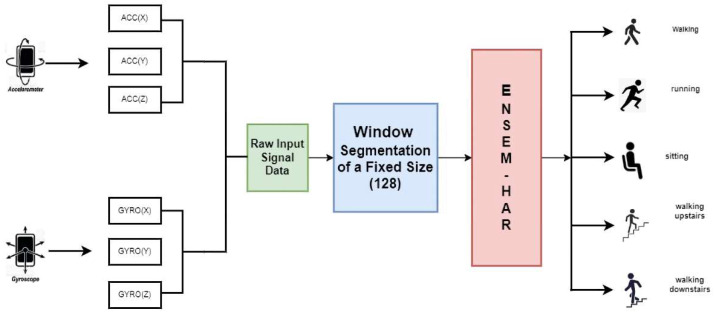
Illustrative overview of collecting the raw data from sensors, pre-processing and window segmentation on that raw data, and finally our proposed Ensem-HAR model for predicting human activities with that pre-processed data.

For conducting trials with sensor-based HAR data, information fusion is an intelligent method based on constructing an ensemble of classifiers, although it is essentially unexplored in this sector [3]. The construction of the ensemble is useful as it takes into account the decisions of multiple models instead of depending on a particular single model. Many classification issues, including HAR, have recently benefited from the use of an ensemble of different models. Mukherjee et al. [4], using various combinations such as ‘majority voting’, ‘sum rule’, ‘score fusion’, etc. between existing deep learning classifiers, was able to enhance overall model performance. Das et al. [5] formed an ensemble from the outputs they obtained from models trained on RGB images and sensor data, and observed a significant enhancement in the performance of their HAR model. The key factor in designing an effective ensemble of classification models is the uniqueness of each of the constituent classifiers, as there is no purpose in using them to construct the ensemble if every constituent wrongly predicts a label and they exhibit identical characteristics in the forecasting an instance [3]. Figure 1 shows an overview of our proposed work.

## 2. Literature Review

HAR is a challenging research topic in the area of computer vision. For a long time, researchers all around the world have been working on developing a near-perfect recognition model. HAR has previously been the subject of a great deal of research. This section focuses primarily on summarizing previous activities taken concerning the datasets chosen here.

Deep learning algorithms are very good at processing time-series signals for feature extraction and classification, taking advantage of local dependencies. Scholars have recently become attracted to the application of profound deep learning methods such as Convolutional Neural Network (CNN), Long Short-Term Memory (LSTM), and hybrid models for better recognition of human activity [6].

In the field of classifying images as well as for forecasting raw time-series signals, CNN models are highly capable. Several scholars have taken full advantage of this by using CNN algorithms to recognise raw inertial data from sensor devices and detect human motion.

Chen and Xue [7] proposed a CNN model that recognizes simple motions based on the three-axial signals collected from accelerometers integrated into a smartphone. To process the signals, they built a CNN model and changed the convolutional kernel accordingly. Ronao and Cho [8] developed a unique CNN design for extracting complicated features that makes use of an exploited (1 × 91 × 14) convolutional layer with a small pooling size (1 × 2–1 × 3). The proposed method has been tested using raw data and the temporal properties of Fast Fourier Transformed signals using the created CNN.

The WISDM project was introduced by Kwapisz et al. [9], who chose 29 users to collect data from. On the WISDM dataset, Quispe et al. employed traditional machine learning-based classifiers K Nearest Neighbor (KNN) [10] and achieved the state-of-the-art results. Table 1 highlights previous measurement research on the WISDM dataset.

Ignatov [13] combined statistical features with CNN to obtain information of raw signals. Wan et al. [14] used CNN in the extraction of the local features of sensor data obtained from smartphone signals, reducing the cost of energy consumption. The impact of signal duration on performance was investigated in this study by adjusting the sliding window up to 1 s. Avilés-Cruz et al. [15] established a deep learning method for classifying and analyzing exclusive user-dependent activity recognition using the CNN model. Table 2 highlights previous measurement research on the PAMAP2 dataset.

To minimize the memory and computational expenses of typical CNNs, Lego filters were employed instead of convolutional filters by Tang et al. [16]. The suggested lightweight model does not need any particular network formation or computer resources, and it improves the efficiency and scalability of the experiments. Similar to [16], Cheng et al. [17] presented a new computer-efficient HAR for mobile and wearable devices which uses conditionally parameterized HAR convolution. Experiments were carried out to demonstrate the efficiency of the larger baseline model network.

The CNN models discussed above are capable enough to attain greater accuracy in the recognition of human activities. An assembly of CNNs with variable layers and filters to eliminate accuracy variations was presented by Zhu et al. [18]. Their model recognizes the confused actions and dynamic activity of persons with fewer training data.

Instead of classifying images with CNN models, LSTM models are very good at predicting raw time-series signal sequences. CNN models use “spatial correlations” to categorize images, whereas an LSTM model classifies time series data by processing a complete sequence of data through a feedback link. Researchers have presented various strategies for LSTM-based HAR models.

Agarwal et al. [22] suggested a lightweight profound learning approach to constructing a HAR with fewer computational resources and less delay, allowing the proposed model to be easily employed in real-world applications. Rashid et al. [23] extended the CNN program [22] and offered a low-power adaptive CNN that is energy-efficient and memorable. Zhao et al. [24] developed a bi-directional residual LSTM architecture with the benefit of combining forward and backwards-looking states and a good- and bad-looking direction in time. The residual link used between the stacked cells prevents the problem of gradient disappearance. Table 3 highlights previous measurement research on the UCI-HAR dataset.

The usual solution for the HAR system is to use mostly local features gathered using heuristic approaches. A profound hybrid approach based on integrating CNN with LSTM has been proposed in a study by Sun et al. [25]. Another important challenge in HAR is analyzing the poorly labelled sensor data to deal with the LSTM model. In order to improve performance with weakly labelled sensor data, Zhou et al. [26] designed a semi-surveyed LSTM learning architecture employing a Deep Q-Network.

Hybrid models are thought to be more effective than standard deep learning models in a variety of situations, including adequate training, perplexing actions, and device placement [27].

Using LSTM models coupled with convolutional layers and global pools, Xia et al. [28] demonstrated a profound hybrid method (GAP). Instead of a conventionally connected layer, GAP was used. Moreover, after GAP, batch standardization was added to accelerate the convergence of the proposed system.

Although CNN, LSTM, and hybrid models are important in HAR problems, CNN-based models have several drawbacks; they require a large quantity of data during their training and are both time- and cost-ineffective. To solve the aforementioned difficulties, Mondal et al. [31] implemented Graph Neural Network (GNN) to transform time-series data into a structural representation of graphs.

He et al. [32] suggested a moderate supervised HAR method that deals with sensor data using ‘recurrent attention learning’. Here, the CNN features are retrieved through multiple iterations using a rewarding method of reinforcement learning. Because it is harder to label a dataset collection for a long and complicated series of actions, Zhu et al. [33] used interim assembly of LSTM. Li et al. [34] proposed a model based on residual block and BiLSTM. Residual block is utilised to extract spatial features from multidimensional signals, and the forward and backward dependencies of the feature sequence are derived using BiLSTM.

In the preceding section, we have addressed the use of CNN, LSTM, and hybrid models in the area of HAR. In this paper, we discuss the deep learning-based paradigm and our construction of an ensemble with four deep learning classifiers designed with CNN, LSTM, and hybrid architectures, which can effectively predict human activities across three conventional benchmark datasets of time series.

## 3. Materials and Methods

We considered the following four deep learning-based models.

1. CNN-net (a 1D CNN model with three levels); 2. CNN-LSTM-net (a 1D CNN model with three levels and an LSTM model); 3. ConvLSTM-net (a time-distributed CNN fed to an LSTM before a dense layer); and 4. StackedLSTM-net (a two-layered LSTM) as our base models. By using these base models, we formed our ensemble, called Ensem-HAR, which is discussed in the following sections. First, we discuss each of the base models in brief and present their diagrams.

### 3.1. CNN-Net Model

**Figure 2:**
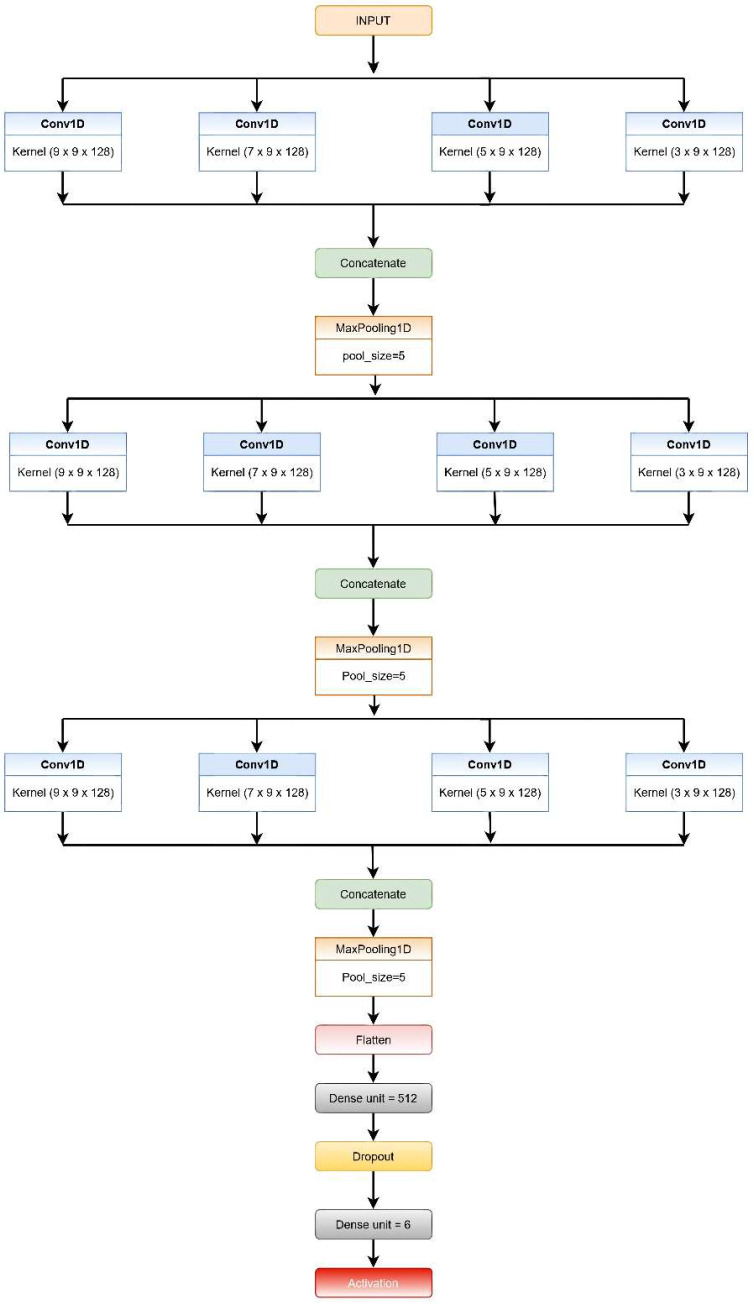
Schematic diagram of the CNN-net model.

A CNN is a deep cascaded artificial neural network (ANN) that is made up of many layers of neural networks, each with a number of neurons. Several critical network layers, such as the “Convolutional Layer”, the “Pooling Layer”, and the “Dense Layer”, play diverse functions in CNNs. The architectural overview of the proposed convolutional neural network model (CNN-net) is depicted in Figure 2.

The CNN model subsequently processes the segmented data. This model comprises three levels of CNN, each consisting of CNN layers, with filters of different kernel sizes in each layer.

- Each CNN level consists of four convolution layers (1D) and incorporates the ReLU activation function, which reduces non-linearity. The filters are of different kernel sizes for each layer, while the number of filters is the same for each level.
- At the very first level, every convolution layer present extracts features from the input windows based on different sized kernels. The features retrieved from each layer in the first level are concatenated, then a max-pooling layer with a five-size pool generates a summary of the extracted features provided by the convolution layers reducing the computation costs.
- The features extracted from the first MaxPool layer are then fed into the second and third set of a four-layered CNN consecutively in a similar fashion. The layers in both the second and third levels have different sizes of kernels, as in the first level; however, the count of filters used in each layer is the same for the respective levels (64 and 32).
- The output from the third MaxPool layer is flattened and fed to the classification layer. This layer is made up of two fully connected (FC) layers that use the SoftMax Activation function on their inputs.
- The addition of dropout after the first layer of the FC layer is performed for regularization, i.e., to minimize the likelihood of overfitting. The Adam optimization method is used by all of the systems for weight updating and loss computation.

### 3.2. CNN-LSTM-Net Model

Long short-term memory networks, or “LSTMs”, are a type of recurrent neural network that can learn long-term dependencies. Their default behaviour is to keep information in their memory for longer periods.

**Figure 3:**
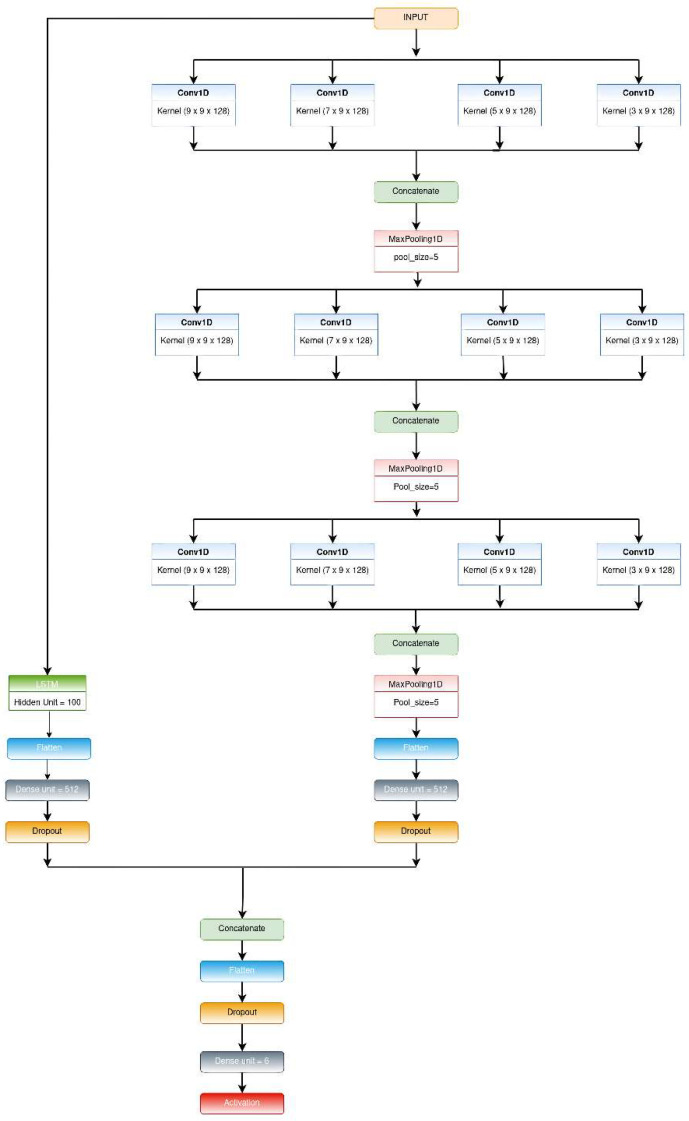
Schematic diagram of the CNN-LSTM-net model.

The mentioned architecture is an extension of the previously proposed model, CNN-net. In this proposed model, we added an LSTM network in parallel with the CNN-net; the features extracted from them were passed through the dense layer and a dropout layer, respectively, then concatenated and fed to the classification layer and SoftMax activation function. The architectural overview of the proposed CNN-LSTM-net is depicted in Figure 3.

### 3.3. ConvLSTM-Net Model

In the ConvLSTM architecture, the CNN layers collect features from input data, while LSTMs facilitate sequence prediction [35]. The ConvLSTM model receives subsets of the main set of input as blocks and extracts features out of each block, then lets the LSTM analyze those features to obtain the prediction. In order to operationalize this concept, we took the approach of dividing each window of *n* time steps into equal-sized sub-sequences for the CNN architecture to process. In this proposed model, we divided each window of 128-time steps into four corresponding sub-sequences of 32 time steps.

We then created a CNN architecture that reads the sequences of 32-time steps with *n* features.

- We wrapped the complete CNN model in a time-distributed layer, allowing it to read in each of the four sub-sequences.
- In the proposed model, we have used three time-distributed layers of the mentioned type, and the output from them was provided to a two-layer stacked LSTM.
- The output obtained from the LSTM layer was forwarded to the classification layer, which was made up of two fully connected (FC)layers that use the SoftMax Activation function on their inputs.
- The number of filters and kernel size of the 1D convolutional layers and the number of hidden units present in the LSTM layers were determined by implementing a random search for a range of values for these parameters.

**Figure 4:**
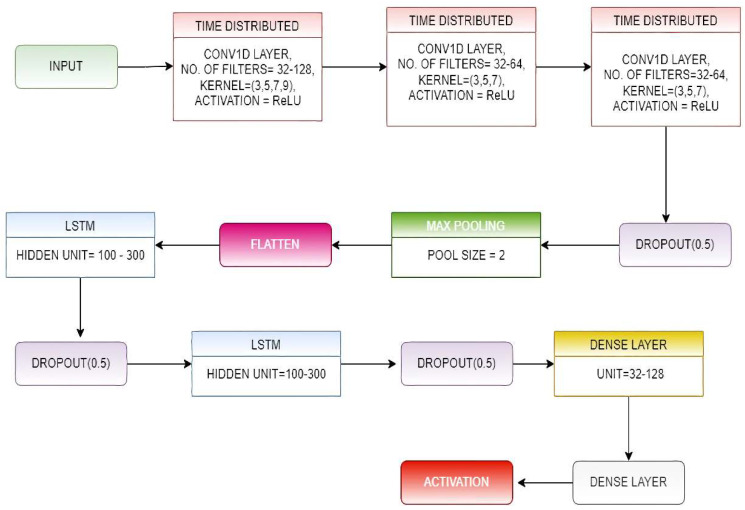
Schematic diagram of the ConvLSTM-Net model.

The overall architecture of the proposed ConvLSTM-net is shown in Figure 4.

### 3.4. StackedLSTM-Net Model

Stacked LSTMs have become a well-established approach for solving difficult sequence prediction challenges in deep learning. The Stacked LSTM architecture is made up of multiple LSTM layers present one after another, all processing data one by one. An LSTM layer above sends a sequence of values to the LSTM layer below as input instead of providing a single value. In our proposed model, the Stacked LSTM model comprises two LSTM layers.

- Each LSTM layer consisted of 128 hidden units and a dropout layer added to reduce overfitting.
- Batch normalization was added after each LSTM layer to standardize the inputs to a layer for each mini-batch.
- The output from the stacked-LSTM was fed to the classification layer, comprising two fully connected (FC) layers, which subject their inputs to the SoftMax activation function.

**Figure 5:**
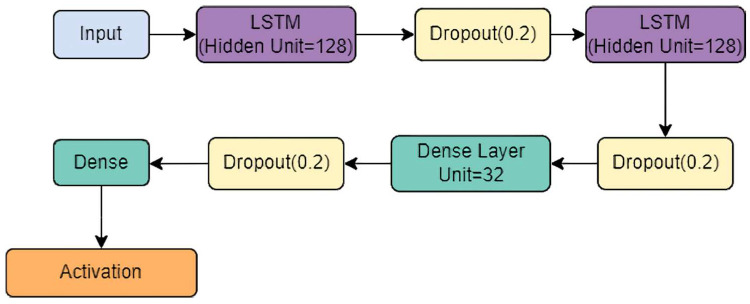
Schematic diagram of the StackedLSTM-net model.

The overall architecture of the proposed StackedLSTM-net is shown in Figure 5.

The predictions from these four foundation models are combined to generate the ensemble. The technique we employed for the ensemble of these base models is called stacking (short for stacked generalization) [36], based on the simple idea that rather than utilizing basic functions (such as hard voting) to combine the predictions of all base models in an ensemble, why not train a machine learning model to do it?

**Figure 6:**
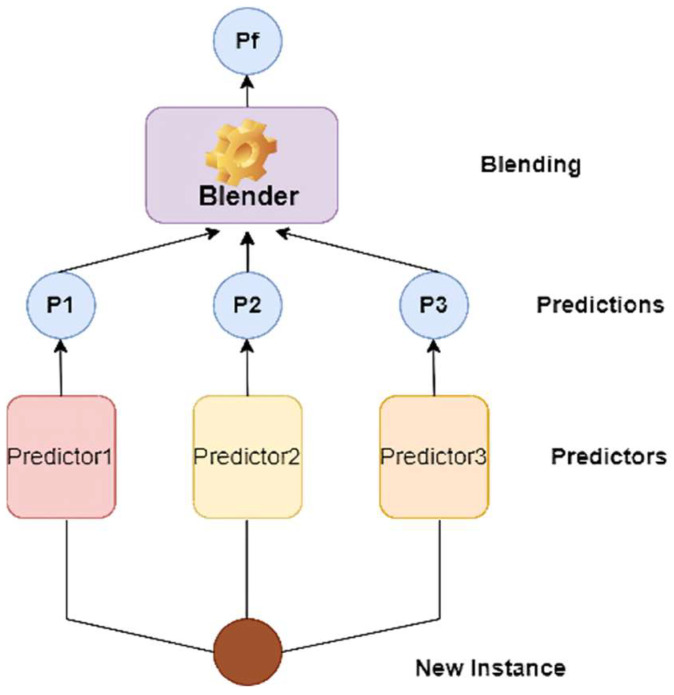
Aggregating predictions using a blending predictor: P1, P2, and P3 are the predictions from all three predictors, respectively, and Pf is the final prediction from the blender.

Figure 6 demonstrates a prediction job performed by such an ensemble on a new instance. The bottom three predictors each predict a different value (P1, P2, P3), and the final predictor (known as a blender or a metalearner) uses these predictions as inputs to generate the final prediction (Pf).

**Figure 7:**
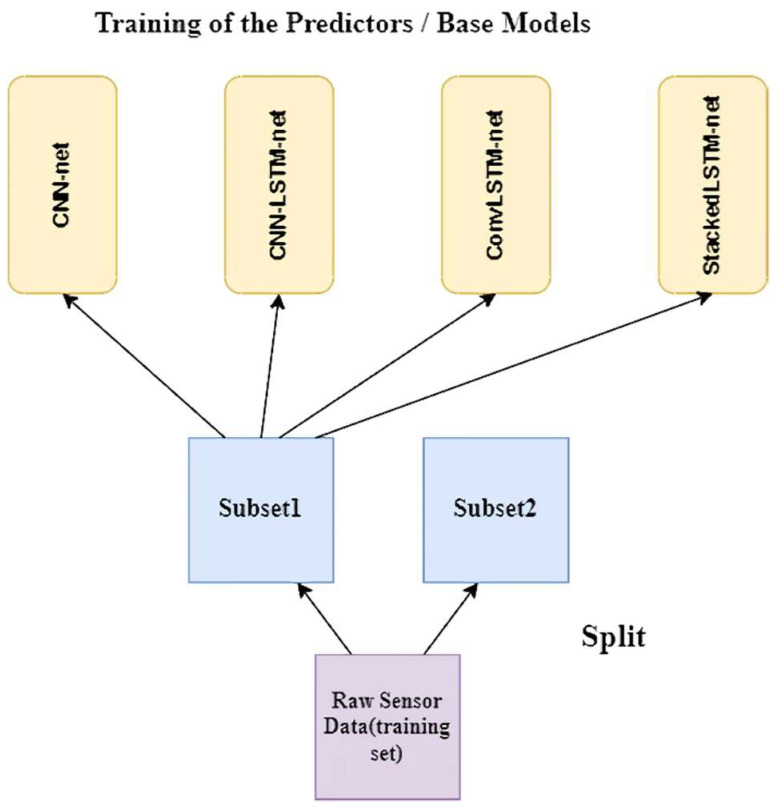
Training the predictors or base models.

Keeping a hold-out set is a standard method for training a blender. The training data set is initially divided into two subsets. Here, we trained the first layer predictors (or base models) with the first subset (see Figure 7).

**Figure 8:**
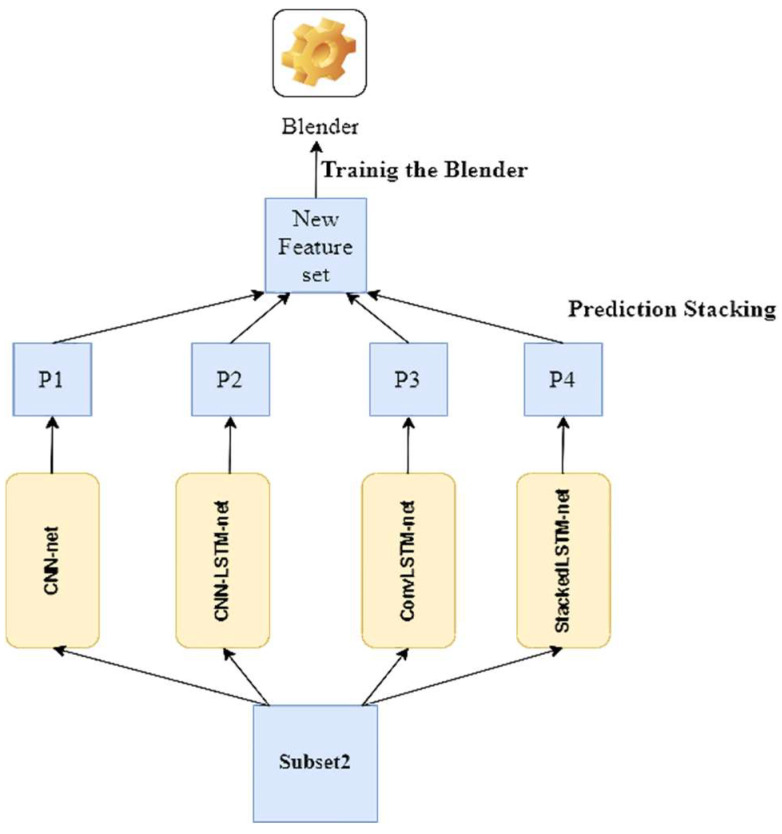
Training the blender.

Then, we take predictions on the second (held-out) set using the first layer predictors. Because the predictors never encountered these events during training, the predictions here are “clean”. Now, these fresh predictions are stacked to make a new feature set, and the blender is trained using this new feature set and the original target values/labels of the held-out set (see Figure 8).

**Figure 9:**
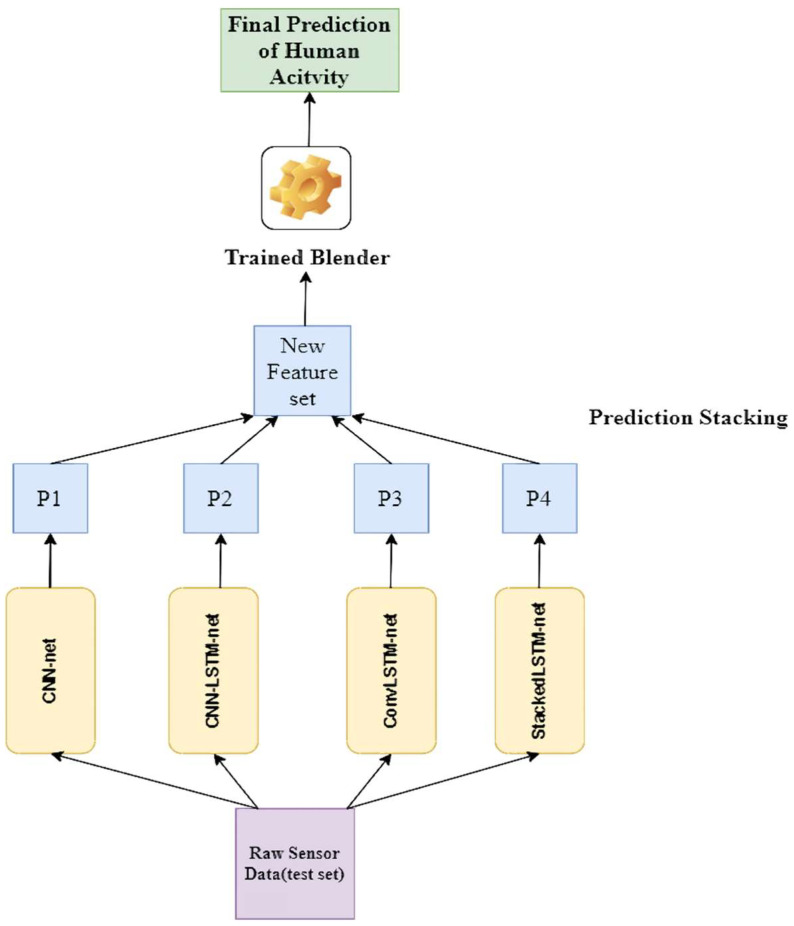
The architectural overview of our proposed Ensem-HAR model.

As the blender is trained (in this study, we used a Random Forest Classifier), the predictions based on the test data are taken sequentially (see Figure 9). This is done by taking predictions from the base models or predictors of the first layer on the test set, then stacking those predictions to make a new feature set; finally, the trained blender provides the final prediction on this stacked feature set.

From the above discussion of how our proposed Ensem-HAR model works, it can be seen that the proposed Ensem-HAR model is not a stand-alone model; instead, it is a combination (technically called an ensemble) of four deep learning classifiers. Thus, the errors made in prediction by any particular classifier can be neutralized by other classifiers in the final ensemble. The main factor to be considered here is that the classifiers should be distinct enough that that they exhibit different characteristics in their predictions, and hence can complement each other’s errors, which in turn lead to higher final recognition accuracy in predicting human activities.

## 4. Result and Analysis

### 4.1. Dataset Description

#### 4.1.1. WISDM Dataset

Kwapisz et al., 2011 [9] created the mentioned dataset by capturing different human activities such as ‘sitting’, ‘walking’, ‘jogging’, ‘standing’, ‘walking downstairs’, and ‘walking upstairs’ with a sampling rate of 20 Hz using an accelerometer integrated with the participants’ smartphones; 36 participants completed the six exercises listed above, and for each exercise, acceleration was recorded along three axes (x, y, z), constituting three features. Then, the raw-sensor data were segmented into fixed-sized windows with 50% overlap (128 readings per window).

#### 4.1.2. PAMAP2 Dataset

A physical activity tracking dataset was developed by A. Reiss and D. Stricker [37] which includes a variety of activities carried out by nine participants. All of the participants were given eighteen different activities to complete (out of which six activities were optional), including ‘rope Jumping’, ‘running’, ‘soccer’, etc. Three sensors placed at different sites on the participants’ bodies were utilized to capture activity-related data. At a sampling rate of 100 Hz (i.e., in each second 100 samples are recorded), a total of 52 features were recorded. In this study, twelve out of the eighteen daily activities were used for experimental purposes. In addition, the researchers segmented the sensor data into fixed-sized windows with a 50-per cent overlap (128 readings per window).

#### 4.1.3. UCI-HAR Dataset

Anguita et al. [38] compiled the mentioned dataset. A total of 30 individuals took part in the study, which included daily human activities such as ‘sitting’, ‘lying’, ‘walking’, ‘standing’, ‘walking upstairs’, and ‘walking downstairs’. Through an accelerometer and a gyroscope installed on subjects’ smartphones, the authors were able to capture linear acceleration and angular velocities along three axes (i.e., x, y, z). At a sampling rate of 50 Hz (i.e., each second, 50 samples are recorded), a total of nine features were captured and with a 50% overlap, the data was segmented into fixed-sized windows (128 readings per window). There are a total of 10,299 samples in the mentioned dataset, which is already segregated according to user ID.

### 4.2. Machine Specification

We performed the training and the testing of the base models and the proposed ensemble of them on a machine equipped with an AMD Ryzen5 2500U CPU, 16Gigs of RAM, and an NVIDIA GeForce GTX 1050 GPU. The machine runs on a Windows 10 operating system with 64 Bits. For the development of the proposed model, we used the Python (3.9), TensorFlow (2.7.0), Keras, and Scikit-learn libraries.

### 4.3. Evaluation Metrics

The dataset was divided into two sets for the evaluation procedure, a training set and a testing set. The model was then adjusted to fit the training set. The prediction is made on the basis of the test set,.

To train each of the base models used in the ensemble, certain hyperparameters were used; for instance, we used 64 as batch size, and the count of epochs was 30. The loss caused in the training was quantified using the categorical cross-entropy, which was then optimized using an efficient gradient-descent technique called the Adam optimizer. In the following discussion, we describe the fundamental performance metrics used in this HAR study.

The performance metrics “Precision”, “Recall”, “F1-Score”, and “*Accuracy*” were used to evaluate our HAR models. First, we defined accuracy, which was estimated by dividing the number of accurately categorized instances by the total number of samples.

*Accuracy =* (TP + TN)/(TP + TN + FP + FN) (1)

TP (True Positive) is the number of correctly categorized records belonging to the positive class, while TN (True Negative) is the number of correctly categorized records belonging to the negative class. FP (False Positive) and FN (False Negative) represent the number of incorrectly categorized records belonging to the positive and the negative classes, respectively.

By the two terms, “*Precision*” and “*Recall*”, we mean the ratio of the number of positive samples classified correctly to the number of samples predicted positive and the number of samples that are actually positive, respectively.

*Precision* = TP/(TP + FP) (2)

*Recall* = TP/(TP + FN) (3)

Another important metric is the F1-measure (*F1-score*), which is a single metric that integrates precision and recall. Therefore, the *F1-score* is more accurate in terms of performance measurement of a model than accuracy. Additionally, as the classes are ranked in importance according to their sample fraction, the F1-score is considered the best choice in cases of class imbalance. The *F1-score* is expressed as follows:

*F1-score* = (2 ∗ Precision ∗ Recall)/(Precision + Recall) (4)

Apart from these evaluation metrics, another important measure of performance in classification models is the “Receiver Operating Characteristic” or ROC curve. It is a graphical representation between the true positive rate (TPR) and false positive rate (FPR) at all levels of classification thresholds. The “Area Under the ROC Curve”, or simply AUC, is the two-dimensional area underneath the ROC curve. Its value lies between 0 and 1. A value of AUC close to 1 indicates that the model is more sensible, while an AUC value of less than 0.5 indicates that the model cannot be considered for making the prediction.

### 4.4. Analysis on Conventional Datasets

#### 4.4.1. Analysis on WISDM Dataset

**Figure 10:**
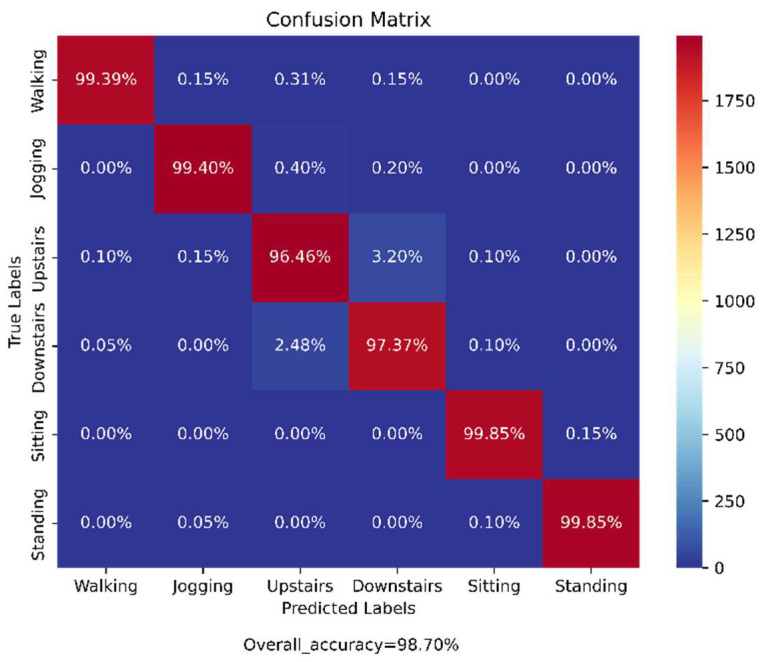
Confusion matrix from the evaluation of our proposed Ensem-HAR model on the WISDM dataset.

**Figure 11:**
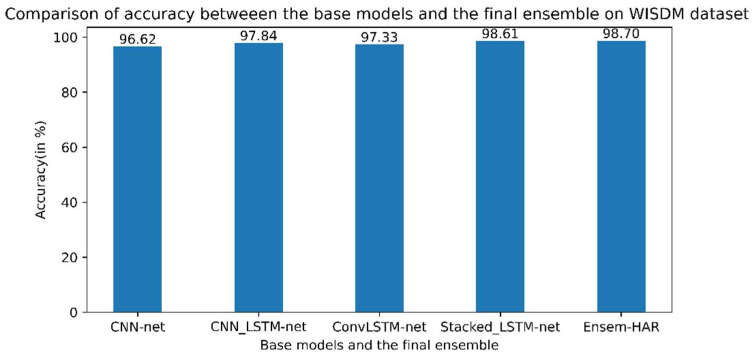
Accuracy (in %) comparison between four base models and Ensem-HAR model on the WISDM dataset.

The samples of the pre-processed WISDM dataset were split into the training (70%) and test (30%) datasets for the training of the previously mentioned base models and then for the testing on the ensemble of those models, respectively. The confusion matrix obtained from the evaluation of the trained proposed model on the test data is shown in Figure 10. According to the obtained confusion matrix, the classification accuracy of our proposed model is more than around 97% for all six activity classes and the overall accuracy is 98.71%, whereas the base models, viz., ‘CNN-net’, ‘CNN-LSTM-net’, ‘ConvLSTM-net’, and ‘StackedLSTM-net’, have accuracies of 96.62%, 97.84%, 97.33%, and 98.61%, respectively, as shown in Figure 11.

From the confusion matrix in Figure 10 it can be seen that except for the ‘Downstairs’ and ‘Upstairs’ activities, all four other activities were perfectly classified (accuracies are almost 100%) by the proposed ensemble of the base models. It can be observed that around 3.2% of samples in the ‘Upstairs’ activity were misclassified to ‘Downstairs’ activity and around 2.4% of samples belonging to ‘Downstairs’ activity were wrongly classified to ‘Upstairs’ activity, as they are opposing kinds of activities.

**Figure 12:**
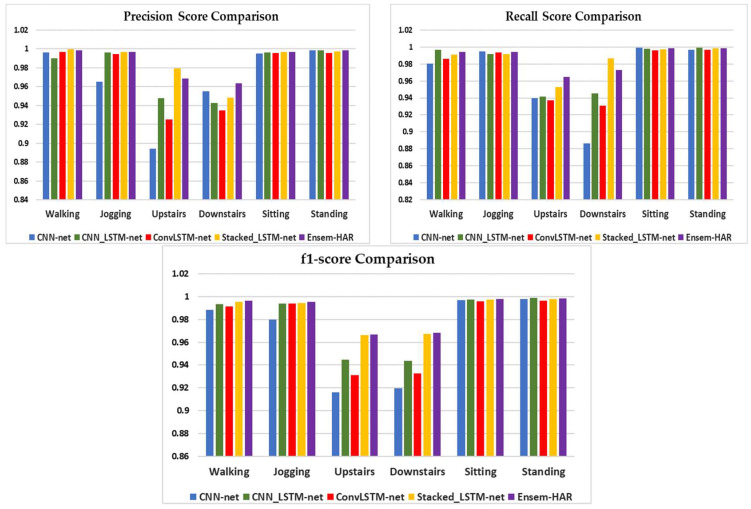
Visual comparison of performances for each activity class in respect of Precision, Recall and F1-score between ‘CNN-net’, ‘CNN-LSTM-net’, ‘ConvLSTM-net’, ‘StackedLSTM-net’, and ‘Ensem-HAR’ models on WISDM dataset.

A visual comparison of the four individual base models’ performance, as well as our proposed Ensem-HAR model, in terms of Precision, Recall and F1-score is shown in Figure 12.

**Figure 13:**
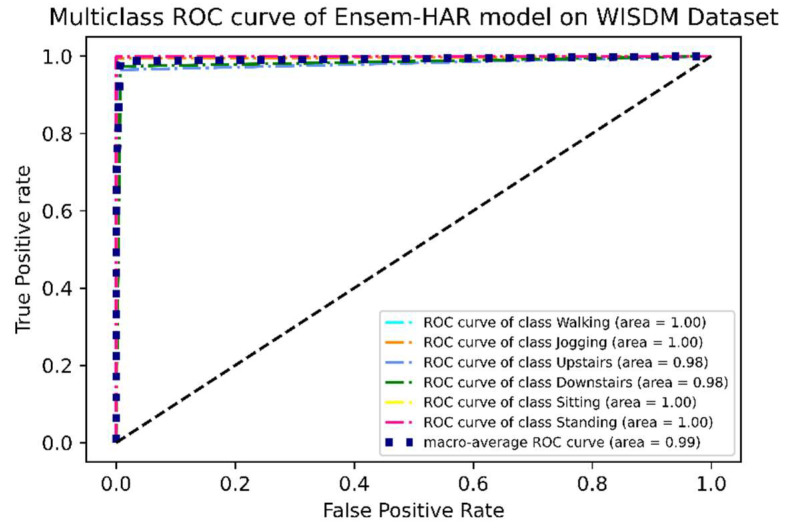
ROC curve (one vs. rest) of the proposed Ensem-HAR model on the WISDM dataset.

It can be observed from the ROC curve shown in Figure 13 that the area under ROC or AUC of every class with respect to the others is almost ‘1′, which indicates that our proposed model was able to classify all activity classes efficiently, as seen earlier using the confusion matrix.

#### 4.4.2. Analysis on PAMAP2 Dataset

**Figure 14:**
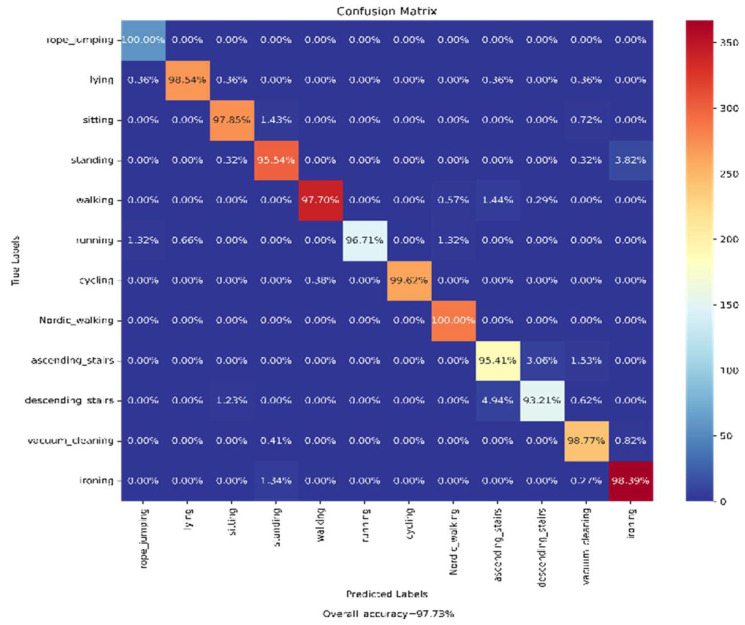
Confusion matrix achieved from the evaluation of our proposed Ensem-HAR model over the PAMAP2 dataset.

**Figure 15:**
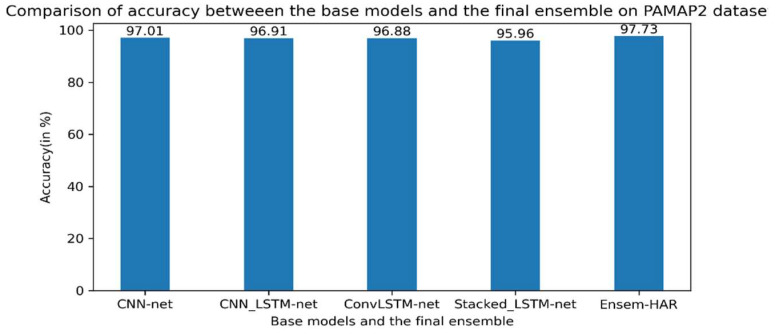
Accuracy (in %) comparison between four base models and Ensem-HAR model on the PAMAP2 dataset.

The samples present in the processed PAMAP2 dataset were split into the training (70%) and test (30%) datasets for the training of the previously mentioned base models and then for testing on the ensemble of those models, respectively. The Confusion Matrix achieved on evaluation of our trained model on the test data is shown in Figure 14. According to the obtained confusion matrix, the classification accuracy of our proposed model is around 96% in ten out of twelve activity classes, excepting two activity classes (‘Ascending_Stairs’ and ’Descending_Stairs’). However, the overall accuracy is 97.73%, where the base models ‘CNN-net’, ‘CNN-LSTM-net’, ‘ConvLSTM-net’, and ‘StackedLSTM-net’ have accuracies of 97.01%, 96.91%, 96.88%, and 95.96%, respectively, as shown in Figure 15.

It can be seen from the confusion matrix shown in Figure 14 that except for two (‘Ascending_Stairs’ and ’Descending_Stairs’), the activities are well classified by the proposed ensemble of the base models. It can be observed that around 4% of samples for both the ‘Ascending_Stairs’ and ’Descending_Stairs’ activities were misclassified, as they are opposing kinds of activities. There was another a misclassification by our proposed model between the ‘Ironing’ and ‘Standing’ activities; around 3% of the sample for ‘Standing’ is misclassified as ‘Ironing’, as both cases involve comparable linear acceleration.

**Figure 16:**
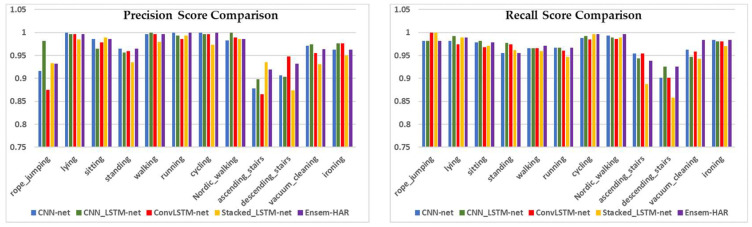

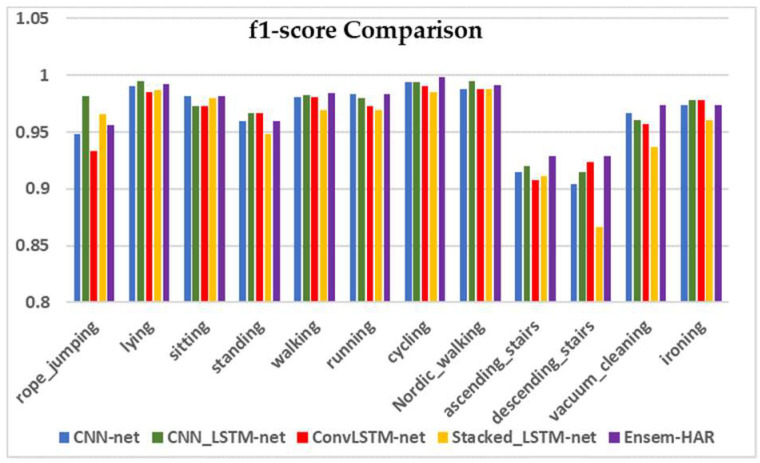
Graphical comparison of performances for each activity class with respect to Precision, Recall, and F1-score between ‘CNN-net’, ‘CNN-LSTM-net’, ‘ConvLSTM-net’, ‘StackedLSTM-net’, and ‘Ensem-HAR’ models on PAMAP2 dataset.

The graphical comparison of the four individual models’ performance and our proposed Ensem-HAR model with respect to Precision, Recall, and F1-score values for each activity class of the PAMAP2 dataset is shown in Figure 16.

**Figure 17:**
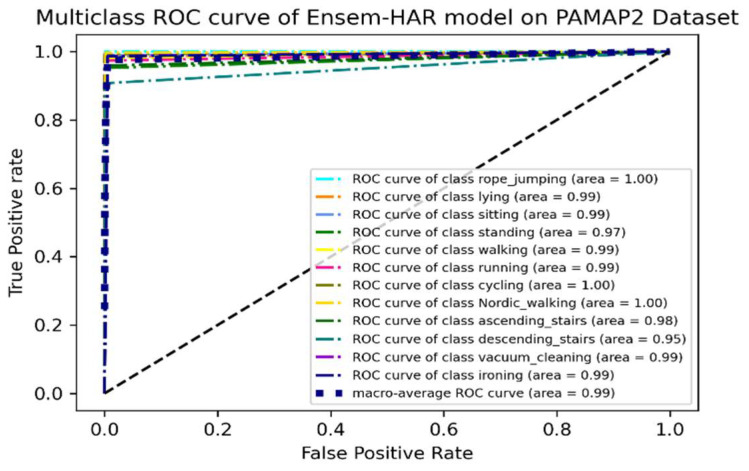
ROC curve (one vs. rest) of the proposed Ensem-HAR model on the PAMAP2 dataset.

It can be observed from the ROC curve shown in Figure 17 that the area under ROC or AUC of every class with respect to the others is almost ‘1′, which indicates that our proposed model was able to classify all activity classes efficiently, as earlier discussed using the confusion matrix.

#### 4.4.3. Analysis on UCI-HAR Dataset

**Figure 18:**
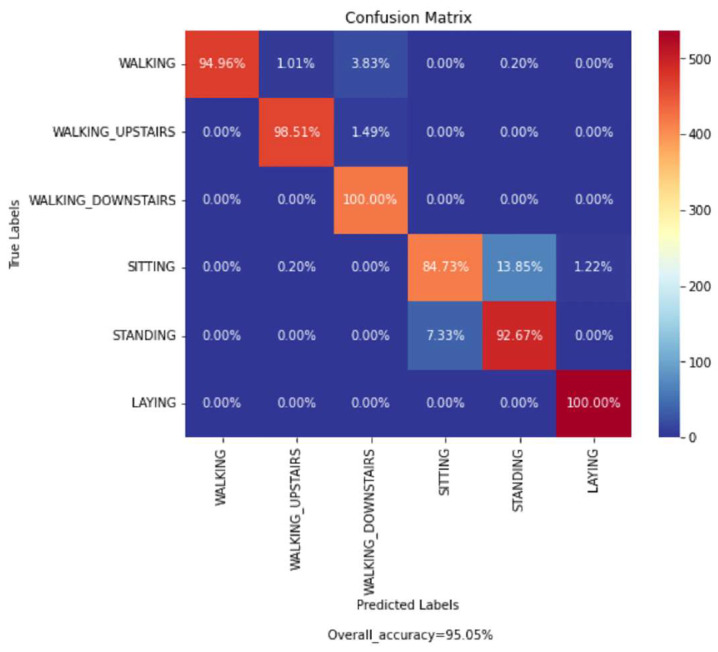
Confusion matrix achieved from the evaluation of our proposed Ensem-HAR model on the UCI-HAR dataset.

**Figure 19:**
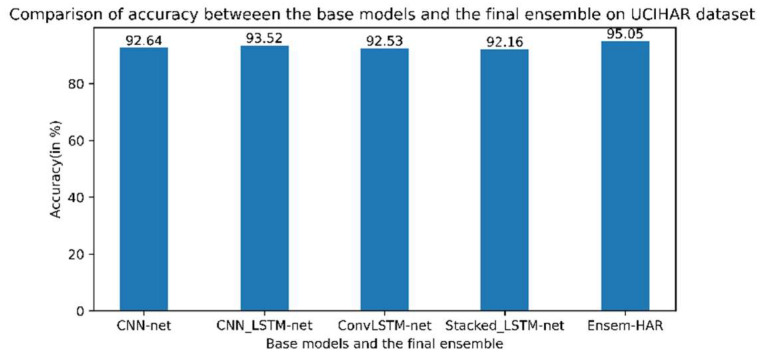
Accuracy (in %) comparison between four base models and Ensem-HAR model on the UCI-HAR dataset.

Out of the total samples present in the UCI-HAR dataset, 7352 samples (training data) were used to train each of the proposed base models and 2947 samples were used as testing data to evaluate the performance of our final ensemble model. The confusion matrix obtained from evaluation of the trained proposed model on the test data is shown in Figure 18. According to the obtained confusion matrix, the classification accuracy of our proposed model is greater than 94% in four out of six activity classes, excepting the activity class ‘Sitting’ and ‘Standing’. However, the overall accuracy is 95.05%, where the base models ‘CNN-net’, ‘CNN-LSTM-net’, ‘ConvLSTM-net’, and ‘StackedLSTM-net’ have accuracies of 92.64%, 93.52%, 92.53%, and 92.16%, respectively, as shown in Figure 19.

From the confusion matrix (Figure 18), it can be observed that except for two activity classes, i.e., ‘Sitting’ and ‘Standing’; all other activity labels are classified fairly well. Due to the comparable nature of linear acceleration, misclassification occurred between these two classes. In addition, our model misclassified samples of ‘Walking’ activity into ‘Walking_Upstairs’ and ‘Walking_Downstairs’ activities, as these acts of ascending and descending while walking had similarity with normal ‘Walking’ for aged persons, as they do these at a slow rate.

**Figure 20:**
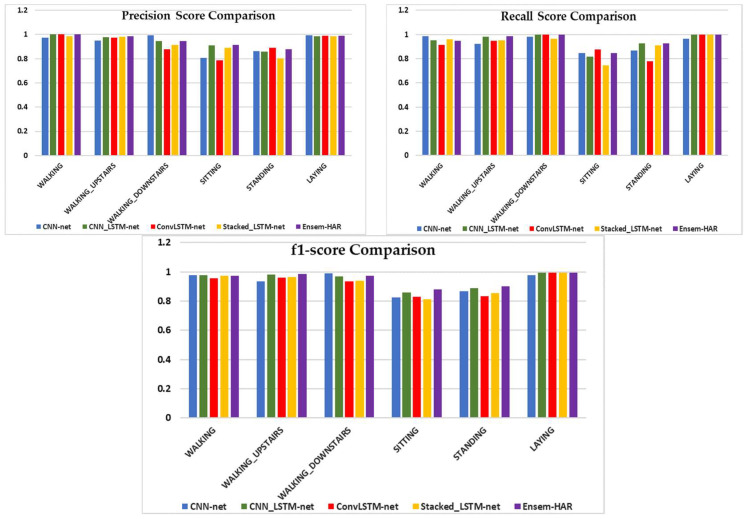
Visual comparison of performance for each activity class in respect of Precision, Recall, and F1-score between ‘CNN-net’, ‘CNN-LSTM-net’, ‘ConvLSTM-net’, ‘StackedLSTM-net’, and ‘Ensem-HAR’ models on the UCI-HAR dataset.

A visual comparison of the four individual models’ performance, as well as our proposed Ensem-HAR model, is shown in Figure 20.

**Figure 21:**
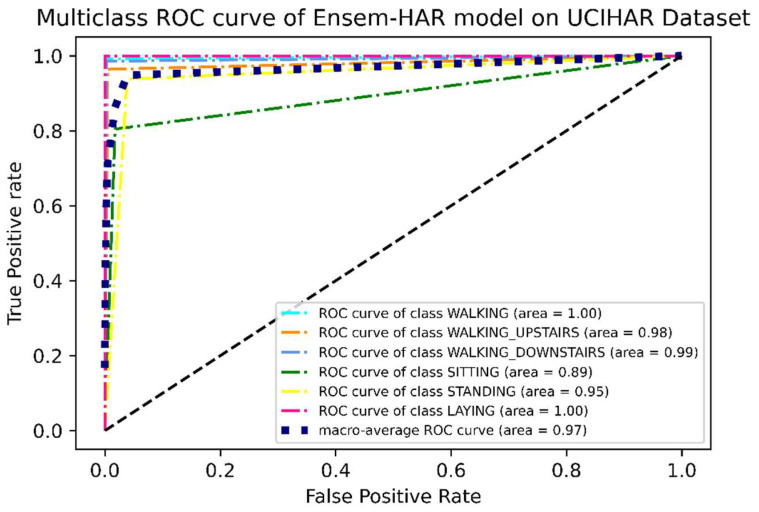
ROC curve (one vs. rest) of the proposed Ensem-HAR model on UCI-HAR dataset.

It can be observed from the ROC curve shown in Figure 21 that, except for two activity classes, i.e., ‘Sitting’ and ‘Standing’, the area under the ROC curve or AUC is almost ‘1′ for the rest of the activity classes, while for these two classes the values are 0.89 and 0.95 respectively. This concludes the observation we made from the confusion matrix (Figure 18), i.e., except for these two activity classes, others have larger accuracies.

### 4.5. Statistical Test

In the previous section, we have performed a detailed analysis of the performance of our proposed model on three benchmark HAR datasets and found that the proposed ensemble of the four base models outperforms each of them in terms of accuracy. For the concrete establishment of the superiority and effectiveness of our proposed ensemble model over the base models, we performed a non-parametric statistical test called the Friedman test [39].

For the Friedman test, we randomly chose ten different subsets, each consisting of 50 samples from the test data for each considered dataset, where all the class labels have equal representation. Then, the classification accuracies of each model over those samples were measured and ranked according to their accuracies and we calculated the mean rank for each model over all the ten samples using the formula

(5) $$R_{j}=\frac{1}{N}\sum_{i=1}^{N}r_{j}^{i}$$

where, $r_{j}^{i}$ is the rank of jth classifier or model for the ith sample. The calculated mean ranks of the classifiers are shown in Table 4.

The null hypothesis ($H_{0}$) states that all the classifiers or models are the same. Therefore, their rank must be equal. For the justification of the null hypothesis, we calculated the value of the Friedman statistic by the following formula [40]:

(6) $$x_{F}^{2}=\frac{12N}{\left(k+1\right)k}\left[\sum_{j}Rj^{2}-\frac{k{\left(k+1\right)}^{2}}{4}\right]$$

where k is the number of classifiers (here, 5) and N is the number of sample datasets (here, 10). The calculated value of the statistic for the three different HAR datasets used in this experiment is shown in Table 5.

**Figure 22:**
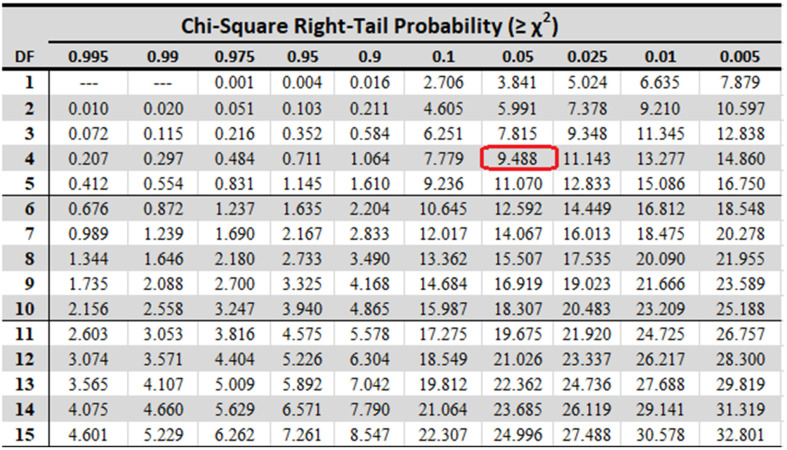
Chi-Square Table.

It can be seen from the Chi-square table (shown in Figure 22) that at k−1 (here 4) degrees of freedom (d.o.f), the standard Friedman static value at significance level 0.05 is found to be 9.49 which is much less than the calculated ones in Table 5. Hence, we can reject the null hypothesis. It can be said from the above experiment that the results achieved by the base models and proposed ensemble model are statistically significant, i.e., not equivalent.

**Figure 23:**
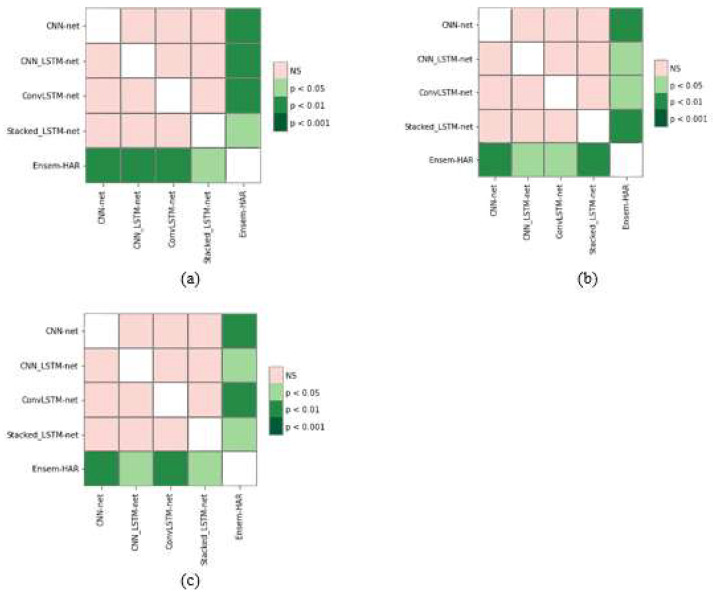
Pairwise Nemenyi score plot generated for: (**a**) WISDM; (**b**) PAMAP2; and (**c**) UCI-HAR datasets.

As it can be seen that the classifiers (or models) are not equivalent, in order to establish the effectiveness of our proposed model we performed a post hoc analysis through the Nemenyi test [40] and calculated the pairwise Nemenyi score, which is the pairwise p-value between two classifiers (or models), and plotted them through a heat map (as illustrated in Figure 23).

From Figure 23, it can be observed that the pairwise p-values between our proposed Ensem-HAR model and the other base models are less than the significance level of 0.05. Hence, it can be concluded that our proposed model is more effective than the four base models, which verifies the effectiveness of the ensemble setup.

For the above-mentioned statistical tests, it should be noted that we randomly considered ten different subsets from the test data. Due to this randomness in selecting the samples, the results of the same test may vary. This means that if the same statistical tests are performed again by selecting random subsets just as before, the accuracies of the base models as well as the final ensemble may change significantly. This can subsequently lead to an alteration in the ranks of the models, and hence the calculated Friedman statistic value may be changed. As a result, the final ensemble model can become statistically insignificant at the significance level of 0.05. Similarly, either for a new HAR dataset other than the datasets used in this experiment or for any other specific application, it should be kept in mind that the proposed Ensem-HAR model may not perform well as the one in this study compared to the four base models.

### 4.6. Performance Comparison to Cutting-Edge HAR Methods

Table 6 shows a comparative study of our proposed Ensem-HAR model with other approaches that have been used for HAR-based problems on the selected benchmark datasets. Based on the comparison of the proposed model to various previous approaches, it can be concluded that our method outperforms the ones listed below. Although there are several approaches mentioned in Table 6, such as U-Net by Zhang et al. [11], which have better accuracy on the UCI-HAR dataset, our model outperforms them in the WISDM dataset. Similarly, the ST-deepHAR proposed by Abdel-Basset et al. [40] has slightly better accuracy for the WISDM dataset in comparison with our proposed Ensem-HAR model; however, the computational complexity of this work is high compared to our proposed Ensem-HAR model.

### 4.7. Performance Comparison with Other Ensemble Methods

Table 7 shows that our proposed ensemble method (Ensem-HAR) is better than several well-known state-of-the-art ensemble techniques, viz., Max Voting, Average (based on the average of class probabilities of each model), and Weighted Average (based on average of class probabilities multiplied by model’s weight) in terms of classification accuracy tested on three HAR datasets.

## 5. Conclusions

In this paper, we have presented an ensemble measurement-based deep learning-based model utilizing four CNN and LSTM-based models called Ensem-HAR for smartphone sensor-based HAR problems. We considered three conventional and publicly available datasets; our proposed model performed well on these datasets and did a commendable job in the prediction of activities with good accuracy. Although in certain cases a high correlation between activities leads to misclassification, it was able to outperformed several recent methods applied to the mentioned datasets. This work, however, has room for improvement. The models we used to construct the ensemble can be selected in such a way that they are slightly distinct from one another. As a result, each component of the ensemble can show more diversity in its traits. This makes the ensemble more accurate. In addition, it is possible to take different measurement approaches to forming the ensemble of the base models. Furthermore, before being fitted into a model, extra work on time-series data processing can be carried out. Each time-series of raw sensor data can be converted into an image or matrix using concepts such as “Gramian Angular Fields (GAF)” and “Markov Transition Fields (MTF)” [43]. Then, transfer learning techniques [44] can be used on those images, or alternatively can be used in models build with CNN and other deep learning measurement architectures [45]. Moreover, it is possible to apply deep temporal Conv-LSTM architecture [46] in order to improve the overall performance of HAR by using both temporal features from sensor data as well as the relationship of sliding windows.

## Figures and Tables

**Table 1:** Performance measurement of some latest HAR approaches on the WISDM dataset.

Author	Year of Publication	Model/Classifier	Accuracy (in %)
Kwapisz et al. [9]	2010	MLP	91.7%
Zhang et al. [11]	2018	U-Net	97%
Quispe et al. [10]	2018	KNN	96.2%
Pienaar et al. [12]	2020	RNN-LSTM	94%

**Table 2:** Performance measurement of the latest HAR approaches on the PAMAP2 dataset.

Author	Year of Publication	Model/Classifier	Accuracy (in %)
Challa et al. [19]	2021	CNN-BiLSTM	94.29%
Dua et al. [20]	2021	CNN-GRU	95.27%
Wan et al. [14]	2020	CNN	91%
Tang et al. [21]	2019	CNN with Lego Bricks with lower Dimensional filter	91.40%

**Table 3:** Performance measurement of the latest HAR approaches on the UCI-HAR dataset.

Author	Year of Publication	Model/Classifier	Accuracy (in %)
Zhao et al. [24]	2018	Residual Bi LSTM	93.6%
Xia et al. [28]	2020	LSTM-CNN	95.78%
Wang and Liu [29]	2020	Hierarchical Deep LSTM	91.65%
Cruciani et al. [30]	2020	CNN	91.98%

**Table 4:** Mean ranks assigned to the four base models and the proposed Ensem-HAR model, according to their accuracies on ten different subsets of each HAR dataset.

Model	Mean Rank of Each Model for Each HAR Dataset		
	WISDM	PAMAP2	UCI-HAR
**CNN-net**	3.55	3.65	3.65
**CNN-LSTM-net**	3.5	3.55	3.45
**ConvLSTM-net**	3.2	3.40	3.60
**StackedLSTM-net**	3.55	3.35	3.15
**Proposed Ensem-HAR**	1.2	1.05	1.15

**Table 5:** Calculated value of Friedman statistic for each HAR dataset.

Dataset	Friedman Statistic Value
WISDM	14.04
PAMAP2	15.39
UCI-HAR	16.47

**Table 6:** Performance Comparison (in %) between several of the latest HAR approaches and our proposed Ensem-HAR model.

Dataset	Author	Year	Model/Classifier	Accuracy	Description
**WISDM**	Pienaar &Malekian	2019	RNN-LSTM [12]	94%	Raw data sampled into fixed-sized windows with 50% overlap
	Zhang et al.	2020	U-Net [11]	97%	Raw data sampled into fixed-sized windows with 50% overlap
	Abdel-Basset et al.	2021	ST-deepHAR [41]	98.9%	Raw data sampled into fixed-sized windows with 50% overlap
	**Bhattacharya et al.**	**2022**	**Ensem-HAR**	**98.70%**	Raw data sampled into fixed-sized windows with 50% overlap, and also oversampling done to remove class imbalance
**PAMAP2**	Wan et al.	2020	CNN [14]	91%	Raw data sampled into fixed-sized windows with 50% overlap
	Challa et al.	2021	CNN-BiLSTM [19]	94.27%	Raw data sampled into fixed-sized windows with 50% overlap
	Dua et al.	2021	CNN-GRU [20]	95.27%	Raw data sampled into fixed-sized windows with 50% overlap
	**Bhattacharya et al.**	**2022**	**Ensem-HAR**	**97.45%**	Raw data sampled into fixed-sized windows with 50% overlap
**UCI-HAR**	Cruciani et al.	2020	CNN [30]	91.98%	70% for training and 30% for testing
	Nair et al.	2018	ED-TCN [42]	94.6%	70% for training and 30% for testing
	Zhang et al.	2020	U-Net [11]	98.4%	70% for training and 30% for testing
	**Bhattacharya et al.**	**2022**	**Ensem-HAR**	**95.05%**	70% for training and 30% for testing

**Table 7:** Performance comparison (measured in terms of accuracy) of our proposed Ensem-HAR model with three state-of-the-art ensemble techniques for three HAR datasets.

Dataset	Ensemble Method	Accuracy
**WISDM**	Max Voting	98.50%
	Average	97.90%
	Weighted Average	98.20%
	**Ensem-HAR**	**98.71%**
**PAMAP2**	Max Voting	97.26%
	Average	97.01%
	Weighted Average	97.07%
	**Ensem-HAR**	**97.73%**
**UCI-HAR**	Max Voting	94.26%
	Average	93.98%
	Weighted Average	94.60%
	**Ensem-HAR**	**95.05%**

## Data Availability

No new data were created or analyzed in this study. Data sharing is not applicable to this article. We have used only publicly available datasets for experimentation.

## Code Availability

All of the source codes related to the proposed work can be found at: https://github.com/Debarshi-Bhattacharya/Ensem_HAR (accessed on 8 April 2022).

## References

[1] Bhattacharya S., Shaw V., Singh P.K., Sarkar R., Bhattacharjee D. (2020). SV-NET: A Deep Learning Approach to Video Based Human Activity Recognition. Proceedings of the International Conference on Soft Computing and Pattern Recognition.

[2] Singh P.K., Kundu S., Adhikary T., Sarkar R., Bhattacharjee D. (2021). Progress of Human Action Recognition Research in the Last Ten Years: A Comprehensive Survey. Arch. Comput. Methods Eng..

[3] Dietterich T.G. Ensemble Methods in Machine Learning. Proceedings of the International Workshop on Multiple Classifier Systems.

[4] Mukherjee D., Mondal R., Singh P.K., Sarkar R., Bhattacharjee D. (2020). EnsemCon-vNet: A Deep Learning approach for Human Activity Recognition Using Smartphone Sensors for Healthcare Applica-tions. Multimed. Tools Appl..

[5] Das A., Sil P., Singh P.K., Bhateja V., Sarkar R. (2020). MMHAR-EnsemNet: A Multi-Modal Human Activity Recognition Model. IEEE Sens. J..

[6] Mondal R., Mukhopadhyay D., Barua S., Singh P.K., Sarkar R., Bhattacharjee D., Nayak J., Naik B., Pelusi D., Das A.K. (2021). A study on smartphone sensor-based Human Activity Recognition using deep learning approaches. Handbook of Computational Intelligence in Biomedical Engineering and Healthcare.

[7] Chen Y., Xue Y. A Deep Learning Approach to Human Activity Recognition Based on Single Accelerometer. Proceedings of the 2015 IEEE International Conference on Systems, Man, and Cybernetics.

[8] Ronao C.A., Cho S.-B. (2016). Human activity recognition with smartphone sensors using deep learning neural networks. Expert Syst. Appl..

[9] Kwapisz J.R., Weiss G., Moore S.A. (2011). Activity recognition using cell phone accelerometers. ACM SIGKDD Explor. Newsl..

[10] Quispe K.G.M., Lima W.S., Batista D.M., Souto E. (2018). MBOSS: A Symbolic Representation of Human Activity Recognition Using Mobile Sensors. Sensors.

[11] Zhang Y., Zhang Y., Zhang Z., Bao J., Song Y. (2020). Human Activity Recognition Based on Time Series Analysis Using U-Net. https://arxiv.org/abs/1809.08113.

[12] Pienaar S.W., Malekian R. Human Activity Recognition using LSTM-RNN Deep Neural Network Architecture. Proceedings of the 2019 IEEE 2nd Wireless Africa Conference (WAC).

[13] Ignatov A. (2018). Real-time human activity recognition from accelerometer data using Convolutional Neural Networks. Appl. Soft Comput..

[14] Wan S., Qi L., Xu X., Tong C., Gu Z. (2019). Deep Learning Models for Real-time Human Activity Recognition with Smartphones. Mob. Netw. Appl..

[15] Avilés-Cruz C., Ferreyra-Ramírez A., Zúñiga-López A., Villegas-Cortéz J. (2019). Coarse-Fine Convolutional Deep-Learning Strategy for Human Activity Recognition. Sensors.

[16] Tang Y., Teng Q., Zhang L., Min F., He J. (2020). Efficient convolutional neural networks with smaller filters for human activity recognition using wearable sensors. arXiv.

[17] Cheng X., Zhang L., Tang Y., Liu Y., Wu H., He J. (2022). Real-Time Human Activity Recognition Using Conditionally Parametrized Convolutions on Mobile and Wearable Devices. IEEE Sens. J..

[18] Zhu R., Xiao Z., Li Y., Yang M., Tan Y., Zhou L., Lin S., Wen H. (2019). Efficient Human Activity Recognition Solving the Confusing Activities Via Deep Ensemble Learning. IEEE Access.

[19] Challa S.K., Kumar A., Semwal V.B. (2021). A multibranch CNN-BiLSTM model for human activity recognition using wearable sensor data. Vis. Comput..

[20] Dua N., Singh S.N., Semwal V.B. (2021). Multi-input CNN-GRU based human activity recognition using wearable sensors. Computing.

[21] Tang Y., Teng Q., Zhang L., Min F., He J. (2020). Layer-Wise Training Convolutional Neural Networks With Smaller Filters for Human Activity Recognition Using Wearable Sensors. IEEE Sens. J..

[22] Agarwal P., Alam M. (2020). A Lightweight Deep Learning Model for Human Activity Recognition on Edge Devices. Procedia Comput. Sci..

[23] Rashid N., Demirel B.U., Al Faruque M.A. (2022). AHAR: Adaptive CNN for Energy-efficient Human Activity Recognition in Low-power Edge Devices. IEEE Internet Things J..

[24] Zhao Y., Yang R., Chevalier G., Xu X., Zhang Z. (2018). Deep Residual Bidir-LSTM for Human Activity Recognition Using Wearable Sensors. Math. Probl. Eng..

[25] Sun J., Fu Y., Li S., He J., Xu C., Tan L. (2018). Sequential Human Activity Recognition Based on Deep Convolutional Network and Extreme Learning Machine Using Wearable Sensors. J. Sens..

[26] Zhou X., Liang W., Wang K.I.-K., Wang H., Yang L.T., Jin Q. (2020). Deep-Learning-Enhanced Human Activity Recognition for Internet of Healthcare Things. IEEE Internet Things J..

[27] Guha R., Khan A.H., Singh P.K., Sarkar R., Bhattacharjee D. (2021). CGA: A new feature selection model for visual human action recognition. Neural Comput. Appl..

[28] Xia K., Huang J., Wang H. (2020). LSTM-CNN Architecture for Human Activity Recognition. IEEE Access.

[29] Wang L., Liu R. (2019). Human Activity Recognition Based on Wearable Sensor Using Hierarchical Deep LSTM Networks. Circuits Syst. Signal Process..

[30] Cruciani F., Vafeiadis A., Nugent C., Cleland I., McCullagh P., Votis K., Giakoumis D., Tzovaras D., Chen L., Hamzaoui R. (2020). Feature learning for Human Activity Recognition using Convolutional Neural Networks. CCF Trans. Pervasive Comput. Interact..

[31] Mondal R., Mukherjee D., Singh P.K., Bhateja V., Sarkar R. (2021). A New Framework for Smartphone Sensor based Human Activity Recognition using Graph Neural Network. IEEE Sens..

[32] He J., Zhang Q., Wang L., Pei L. (2018). Weakly Supervised Human Activity Recognition From Wearable Sensors by Recurrent Attention Learning. IEEE Sens. J..

[33] Zhu Q., Chen Z., Soh Y.C. (2018). A Novel Semisupervised Deep Learning Method for Human Activity Recognition. IEEE Trans. Ind. Inform..

[34] Li Y., Wang L. (2022). Human Activity Recognition Based on Residual Network and BiLSTM. Sensors.

[35] Shi X., Chen Z., Wang H., Yeung D.Y., Wong W.K., Woo W.C. (2015). Convolutional LSTM Network: A Machine Learning Approach for Precipitation Nowcasting. Adv. Neural Inf. Processing Syst..

[36] Wolpert D.H. (1992). Stacked generalization. Neural Netw..

[37] Reiss A., Stricker D. Introducing a New Benchmarked Dataset for Activity Monitoring. Proceedings of the 2012 16th International Symposium on Wearable Computers.

[38] Anguita D., Ghio A., Oneto L., Parra-Llanas X., Reyes-Ortiz J. A public domain dataset for human activity recognition using smartphones. Proceedings of the 21th International European Symposium on Artificial Neural Networks, Computational Intelligence and Machine Learning.

[39] Singh P.K., Sarkar R., Nasipuri M. (2016). Significance of non-parametric statistical tests for comparison of classifiers over multiple datasets. Int. J. Comput. Sci. Math..

[40] Singh P.K., Sarkar R., Nasipuri M. (2015). Statistical Validation of multiple classifiers over multiple datasets in the field of pattern recognition. Int. J. Appl. Pattern Recognit..

[41] Abdel-Basset M., Hawash H., Chakrabortty R.K., Ryan M., Elhoseny M., Song H. (2020). ST-DeepHAR: Deep Learning Model for Human Activity Recognition in IoHT Applications. IEEE Internet Things J..

[42] Nair N., Thomas C., Jayagopi D.B. Human Activity Recognition Using Temporal Convolutional Network. Proceedings of the 5th international Workshop on Sensor-based Activity Recognition and Interaction.

[43] Wang Z., Oates T. (2015). Encoding time series as images for visual inspection and classification using tiled convolutional neural networks. AAAI Workshop-Tech. Rep..

[44] Chakraborty S., Mondal R., Singh P.K., Sarkar R., Bhattacharjee D. (2021). Transfer learning with fine tuning for human action recognition from still images. Multimed. ToolsAppl..

[45] Banerjee A., Bhattacharya R., Bhateja V., Singh P.K., Lay-Ekuakille A., Sarkar R. (2022). COFE-Net: An ensemble strategy for Computer-Aided Detection for COVID-19. Measurement.

[46] Noor M.H.M., Tan S.Y., Ab Wahab M.N. (2022). Deep Temporal Conv-LSTM for Activity Recognition. Neural Process. Lett..

